# The dominant component of strong π-hole interactions: electrostatic attraction versus charge transfer

**DOI:** 10.1107/S2052252523001720

**Published:** 2023-03-01

**Authors:** Aleksander Shkurenko

**Affiliations:** a Advanced Membranes and Porous Materials Center, King Abdullah University of Science and Technology, Thuwal 23955, Saudi Arabia

**Keywords:** π-hole interactions, charge transfer, quinone, charge density, Atoms In Molecule analysis

## Abstract

A long-asked question concerning the dominant component of strong π-hole interactions is addressed.

An article in this issue of **IUCrJ** (Milašinović *et al.*, 2023 ▸) addresses a long-asked question concerning the dominant component of strong π-hole interactions.

A deep understanding of intra- and intermolecular non-covalent interactions is crucial for progress in many areas of supramolecular chemistry, especially in the field of molecular recognition and self-assembly, which is the basis of *e.g.* drug design and crystal engineering. By pondering the nature of non-covalent interactions, quantum theory leads us to the conclusion that the interactions within and between such chemical entities as molecules, ions *etc*. are Coulombic, and include electrostatic, polarization, dispersion and charge transfer contributions. Theoretical calculations and crystallographic studies (Politzer *et al.*, 2021 ▸; Stevens, 1979 ▸; Batsanov, 2000 ▸) show a non-uniform electron density distribution on an atom in a molecule. Obviously, the region with a higher electron density is located at the covalent bond, while a region of lower electronic density is at the side opposite to the covalent bond. The difference in electron density between the regions depends on atomic polarizability and is most notable for covalently bonded atoms of Groups 14–17 (Grabowski, 2017 ▸). If an atom participates in a multiple-bond formation, *e.g.* double or triple, the electron-depleted areas are perpendicular to the main molecule. Depending on the positive region location, it is usually defined as a σ-, π-, lone-pair *n*-, or radical *R*·-hole. These electron density holes are responsible for the formation of attractive interactions with any electron-rich site (a lone pair or a π-system of a molecule, an anion *etc*.). Depending on the atoms on which the hole is observed, the most studied kinds of interactions are called hydrogen bonding, and tetrel, pnictogen, chalcogen and halogen interactions.

Among all the above-mentioned hole interactions, the *n*···π-hole one has been attracting particular attention during the past decade for several reasons: (1) significant polarizability of the π-electrons, especially those in conjugated systems, makes estimation of the correct whole system energy hard from the calculational point of view; (2) the important role of the *n*···π-hole interactions in biochemistry (Newberry & Raines, 2017 ▸; Singh & Das, 2015 ▸; Lucas *et al.*, 2016 ▸; Bauzá *et al.*, 2012 ▸); (3) possible applications, *e.g.* in crystal engineering (Chopra, 2018 ▸; Bauzá *et al.*, 2016 ▸; Seth *et al.*, 2018 ▸; Bauzá *et al.*, 2019 ▸), drug design (Singh & Das, 2015 ▸) and molecular recognition (Wang & Wang, 2013 ▸). It is understandable that aromatic rings with multiple electron-withdrawing substituents are the most convenient model molecules due to significant π-hole(s); therefore, they can form the strongest *n*···π-hole interaction(s) (which is the most promising for possible applications) and this is exactly what is observed in most of the cases studied. Recently, tetrahalogenated quinone rings have revealed very positive π-holes (Jalilov *et al.*, 2020 ▸). On the other hand, a perfect electron-reach chemical entity for forming as strong as possible *n*···π-hole interaction is the iodide anion. Indeed, π-hole interactions between iodide and quinone are well known (Molčanov *et al.*, 2018 ▸, 2019 ▸; Milašinović & Molčanov, 2021 ▸) and such quinone–iodide co-crystals are black and opaque, while the crystals containing neutral quinones are light brown and transparent. Both the short quinone–iodide distances and the colour of the co-crystals suggest possible charge transfer from the iodide to the quinone ring. The computational study (Molčanov *et al.*, 2018 ▸) shows a significant discrepancy in the estimated interaction energy: 18 (1) kcal mol^−1^ and 8(2) kcal mol^−1^ calculated by DFT (M06-2X functional) and *ab initio* perturbational MP2 methods, respectively. Moreover, the plane-wave DFT calculations suggest around 30% charge transfer, whereas the single-crystal X-ray diffraction studies reveal proper quinone moieties geometry, not a semi­quinone-like distortion, expected if the moieties have a partial negative charge (Molčanov *et al.*, 2018 ▸; Milašinović & Molčanov, 2021 ▸). Therefore, to gain more insight into the nature of iodide-quinone *n*···π-hole interaction, further systematic studies were needed.

In this issue, Milašinović *et al.* (2023 ▸) have finally found the answer to the question: in this case is the main component of *n*···π-hole interaction electrostatic attraction or charge transfer? For this purpose, the authors synthesized a co-crystal of (3-Cl-*N*-MePy)_2_I_2_·Br_4_Q and grew single crystals suitable for X-ray diffraction studies. The dark colour and opacity of the crystals impede optical spectroscopy studies. The strong absorption forced the use of short-wave synchrotron radiation (0.62009 Å). The high-resolution single-crystal X-ray diffraction data (up to a maximum θ of 40.9°, *d* = 0.475 Å) enabled multipolar refinement and experimental topological analysis of the electron density distribution in the crystal structure. The iodide–quinone *n*···π-hole interaction (Fig. 1 ▸) is responsible for a centrosymmetric sandwich-like I^−^⋯Br_4_Q⋯I^−^ unit formation. The distance of the iodide from the plane of the quinone ring (3.727 Å) is in the range of 3.49–3.81 Å previously observed for the I^−^⋯Br_4_Q⋯I^−^ unit (Molčanov *et al.*, 2018 ▸, 2019 ▸; Milašinović & Molčanov, 2021 ▸). A couple of inversion-related 3-Cl-*N*-MePy cations with antiparallel C–Cl bonds are formed due to π–π stacking interaction with an interplanar separation of 3.388 Å. The cation couples and I^−^⋯Br_4_Q⋯I^−^ unit packing in the crystal structure corresponds to the CsCl structure type. Naturally, the structure is dominated by cation–anion electrostatic interactions. In addition to the iodide–quinone *n*···π-hole interactions and the π–π stacking interactions, the structure is stabilized by halogen and hydrogen bonding. The Hirshfeld surface and Voronoi–Dirichlet polyhedral surface analyses for the Br_4_Q molecule reveal that C···I contact areas of 7.4% and 3.5%, respectively, are in the range of typical values observed for iodide–quinone *n*···π-hole interactions (Milašinović & Molčanov, 2021 ▸). The periodic DFT calculations successfully reproduced the experimental electron density distribution and confirmed that the iodide–quinone *n*···π-hole interaction is predominantly electrostatic with a significant dispersion contribution and minor charge transfer from a lone pair of the iodide into an empty π*-orbital of the quinone (*n* → π*) of 9 (2)%.

Functional materials design based on *n*···π-hole interactions is still a long way off, but the present study may be a good starting point for material property design. The work by Milašinović *et al.* provides a state-of-the-art example of a non-covalent interaction study combining experimental crystal structure and electron density distribution determination and theoretical calculations. Surprisingly, the discussed iodide–quinone *n*···π-hole interaction of 11.16 kcal mol^−1^ surpasses most hydrogen bonds. Therefore, the major benefits should be clearly achievable if the chemical nature of the quinone and the nucleophile are chosen with proper management of strength and directionality.

## Figures and Tables

**Figure 1:**
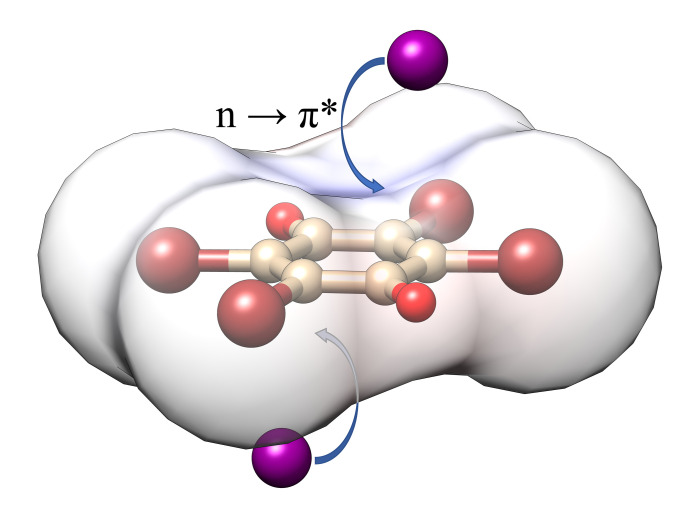
Iodide–quinone *n*···π-hole interactions in the co-crystal of 3-chloro-*N*-methyl­pyridinium iodide with tetra­bromo­quinone .
